# Association between the sinus microbiota with eosinophilic inflammation and prognosis in chronic rhinosinusitis with nasal polyps

**DOI:** 10.1038/s12276-020-0458-1

**Published:** 2020-06-29

**Authors:** Ji Heui Kim, Sung Hee Kim, Ji Youn Lim, Doyeon Kim, In Seong Jeong, Dong Kyu Lee, Yong Ju Jang

**Affiliations:** 1 Department of Otorhinolaryngology–Head and Neck Surgery, Asan Medical Center, University of Ulsan College of Medicine, Seoul, Republic of Korea; 2 Department of Otorhinolaryngology–Head and Neck Surgery, National Medical Center, Seoul, Republic of Korea

**Keywords:** Respiratory tract diseases, Innate immunity, Respiratory tract diseases, Innate immunity

## Abstract

Dysbiosis of the sinus microbiome affects the pathophysiology of chronic rhinosinusitis with nasal polyps (CRSwNPs). We investigated whether the sinus microbiota in CRSwNPs is associated with eosinophilic inflammation, especially in relation to innate lymphoid cells (ILCs), prognosis, and serum extracellular vesicles (EVs). Middle meatal swabs and serum from 31 CRSwNPs patients and six healthy controls were analyzed by 16S ribosomal RNA sequencing. ILC2s and cytokines from sinonasal tissues were measured by flow cytometry and ELISA, respectively. The relative abundances (RAs) of bacteria were compared based on eosinophilic inflammation and surgical outcome. The correlations between sinus bacteria and ILC2s, cytokines, and serum EVs were analyzed. The compositions of sinus bacteria were different between groups at the genus level. In eosinophilic CRSwNPs patients, the RA of *Anaerococcus* was significantly decreased (*P* = 0.010), whereas that of *Lachnoclostridium* was significantly increased (*P* = 0.038) compared with that in controls. The RA of *Lachnoclostridium* showed a significant positive correlation with interleukin (IL)-5-producing ILC2 populations (*R* = 0.340, *P* = 0.049), whereas the RA of *Anaerococcus* showed a negative correlation with IL-5-producing ILC2 populations (*R* = −0.332, *P* = 0.055). The RAs of *Corynebacterium*, *Anaerococcus*, and *Tepidimonas* were significantly decreased in patients with suboptimal outcomes compared with those in patients with optimal outcomes and control subjects. Some sinus bacteria and serum EVs showed positive correlations. CRSwNPs patients showed distinct microbiota compositions based on eosinophilic inflammation in relation to ILC2s and surgical outcome. These findings support a relationship between the microbiota and the host immune response in CRSwNPs.

## Sinus disease: nasal bacteria linked to manifestations of chronic disease

Microbes living in the sinonasal cavity seem to affect the immune responses and clinical outcomes of patients with an inflammatory disease that can cause lasting pain, pressure, and swelling in the sinuses. Ji Heui Kim from the Asan Medical Center in Seoul, South Korea, and colleagues collected nasal swabs and blood from 31 people suffering from chronic rhinosinusitis with nasal polyps and from six healthy control individuals. They identified several specific bacterial groups whose presence or absence was associated with the abundance of certain white blood cell populations, a sign of inflammation. Some of these groups were linked to patient outcomes after nasal surgery but additional groups were also observed. The findings could help lead to new treatment strategies or diagnostic biomarkers for patients with this sinus disorder.

## Introduction

Chronic rhinosinusitis (CRS) is a multifactorial chronic upper airway inflammatory disease. Putative pathological factors include changes in the microbiota, an imbalance of the local or systemic immune system, allergens, toxins, and genetic predisposition^1–3^. Several studies have reported that the microbiota may affect the pathophysiology of CRS. In general, a decrease in microbial diversity and an increase in the total number of microorganisms have been reported in patients with CRS^4,5^. Therefore, dysbiosis of the microbiota could be the driving force of CRS.

Immunologic features associated with the nature or composition of the sinus microbiota could partially explain the heterogeneity of CRS. CRS with nasal polyps (CRSwNPs) is generally classified as eosinophilic or noneosinophilic, which show distinct immunological features. Eosinophilic polyps are associated with the type 2 immune response, while noneosinophilic polyps are associated with type 1 and 17 immune responses^6^. When a CRS patient cluster was divided into distinct subgroups according to the specific pattern of bacterial cocolonization, each subgroup was associated with a host immune response. Some subgroups exhibited increased expression of the *interleukin 6*, *tumor necrosis factor*, *IL8*, and *IL10* genes, whereas other subgroups were associated with induced *IL5* gene expression^7^. Innate lymphoid cells (ILCs) are a recently identified innate lymphocyte population. Group 2 ILCs (ILC2s) secrete type 2 cytokines, such as IL-5 and IL-13, and contribute to the pathogenesis of CRSwNPs, particularly in eosinophilic tissue inflammation^8^. ILCs interact with bacteria and coordinate host–bacteria relationships that are associated with the pathogenesis and progression of numerous chronic human infectious, inflammatory, and metabolic diseases^9^. We speculated that in CRSwNPs, ILCs could interact with the microbiota and influence eosinophil inflammation.

Bacteria secrete extracellular vesicles (EVs), including transmembrane proteins, cytosolic proteins, lipids, and nucleic acids. EVs play a role in immune and airway epithelial cell communication and can induce immune responses in allergic diseases. EVs can be detected in the blood circulation far from the local disease site^10–13^. In nasal lavage fluid from CRS patients, the composition of EVs secreted from microbiota has been positively correlated with bacterial composition^14^. We hypothesized that paranasal sinus bacteria-derived EVs can be detected in the blood. However, the relationship between sinus bacteria and serum EVs is unknown.

In the present study, the sinus microbiota in specimens from middle meatus swabs from CRSwNPs patients was analyzed based on eosinophilic vs. noneosinophilic inflammation. In addition, correlations were analyzed between sinus bacteria and ILCs and cytokines from tissues and between sinus bacteria and serum EVs.

## Materials and methods

### Study populations

This study was conducted in accordance with the Declaration of Helsinki and approved by the Institutional Review Board of Asan Medical Center (2018–0121). All participants provided written informed consent.

Thirty-one adult CRSwNP patients, including 21 eosinophilic CRSwNPs patients and 10 noneosinophilic CRSwNPs patients, and 6 healthy controls were recruited from the Department of Otorhinolaryngology–Head and Neck Surgery, Asan Medical Center, between February 2018 and December 2018. CRSwNPs was diagnosed based on the established guideline criteria of the European Position Paper on Rhinosinusitis and Nasal Polyps (2012), considering the history, nasal endoscopy findings, and computed tomography (CT) images of the paranasal sinuses^2^. Eosinophilic and noneosinophilic CRSwNPs were defined based on the proportion of eosinophils >10% and <10%, respectively, of the total inflammatory cells in the NP tissue^15,16^. Subjects with antrochoanal polyps, fungal sinusitis, allergic fungal sinusitis, aspirin-exacerbated respiratory disease, immunodeficiency, pregnancy, cystic fibrosis, or primary ciliary dyskinesia were not included. All patients refrained from the use of decongestants, antibiotics, and systemic corticosteroids for at least 4 weeks prior to surgery, did not have a history of acute respiratory infection for 4 weeks before surgery, and did not have medical comorbidities that included diabetes mellitus, hypertension, or hyperlipidemia. Demographic and clinical variables recorded for each subject were age, sex, ethnicity, smoking history, allergy, asthma, antibiotic use in the past 6 months, saline rinse use, and topical steroid use in the preceding 4 weeks.

Atopic status was evaluated using a skin prick test (Allergopharma; Hermann-Körner-Straße, Reinbeck, Germany), and specific IgE antibodies against various common inhalant allergens were detected with ImmunoCAP™ tests (Thermo‐Fisher Scientific, Waltham, MA, USA). Asthma and aspirin intolerance were diagnosed based on medical history. Preoperative Lund–Kennedy endoscopy score, polyp grading according to the Davos classification, Lund–Mackay CT scores, and sinonasal outcome test (SNOT-22) scores were evaluated^2^. Control subjects >18 years of age underwent septoplasty for nasal obstruction without any other inflammatory nasal diseases, such as allergic rhinitis or sinusitis, evident on CT scan.

After at least 6 months, CRSwNP patients were examined to determine changes in Lund–Kennedy endoscopy score, the need for revision of sinus procedure(s), and the use of additional courses of antibiotics or systemic steroids after routine standard perioperative administration. The optimal outcome was defined as a reduction in endoscopy scores >50%, no requirement for revision of surgical treatment, and no further use of antibiotics and systemic steroids before the 6-month follow-up visit^17^.

Swab samples (Isohelix DNA swab, Cell Projects Ltd., Maidstone, Kent, UK) for DNA extraction were obtained using an endoscopic guide from the middle meatus and rotated at least five full turns until saturated. The samples were placed on ice upon collection and frozen within an hour at –80 °C until DNA extraction. Care was taken to avoid contamination from the anterior nasal cavity during swabbing. NP tissues from CRSwNP patients and middle turbinate tissues from controls were harvested after swabbing. Blood samples were collected from the median cubital vein after overnight fasting and were transferred into serum separation tubes (BD, Franklin Lakes, NJ, USA). Serum was separated from the blood and frozen at −80 °C until DNA extraction.

### DNA extraction from samples

Human swab samples were vortexed after dilution in 800 µl of phosphate-buffered saline (PBS, Welgene, Gyeongsan, South Korea). After centrifugation at 10,000 × *g* for 1 min at 4 °C, the pellets of swab samples containing bacterial cells were heated for 40 min at 100 °C to extract DNA from the bacterial cells. To eliminate remaining floating particles and debris, the supernatants were collected after centrifugation at 13,000 rpm for 30 min at 4 °C. For serum samples, floating particles were precipitated by centrifugation at 10,000 × *g* for 1 min at 4 °C, after which the supernatants were mixed with PBS. After centrifugation, bacteria and foreign particles were thoroughly eliminated by sterilizing the supernatants through a 0.22-μm filter. Next, the supernatants were boiled for 40 min at 100 °C to extract DNA from EVs and centrifuged at 13,000 rpm for 30 min at 4 °C. The supernatants were collected^18,19^. After this preprocessing, DNA from sinus bacteria and serum bacterial EVs was extracted using a MO BIO PowerSoil DNA Isolation Kit (QIAGEN, Hilden, Germany) and quantified using the QIAxpert system (QIAGEN).

### Bacterial metagenomic analysis using DNA from human samples

Bacterial genomic DNA was amplified with the 16S_V3_F (5′-TCGTCGGCAGCGTCAGATGTGTATAAGAGACAGCCTACGGGNGGCWGCAG-3′) and 16S_V4_R (5′-GTCTCGTGGGCTCGGAGATGTGTATAAGAGACAGGACTACHVGGGTATCTAATCC-3′) primers specific for the V3–V4 hypervariable regions of the 16S ribosomal RNA gene. The libraries were prepared using PCR products according to the MiSeq system guide (Illumina, San Diego, CA, USA) and quantified using the QIAxpert system (QIAGEN). Each amplicon was then quantified, the equimolar ratio was obtained, amplicons were pooled, and they were sequenced on a MiSeq device (Illumina) according to the manufacturer’s recommendations.

### Analysis of the bacterial microbiota composition

Paired-end reads that matched the adapter sequences were trimmed using cutadapt version 1.1.6^20^. The resulting FASTQ files containing paired-end reads were merged with The Context-Aware Scheme for Paired-End Read tool, and they were quality filtered using the Phred (Q) score based on previously described criteria^21,22^. Reads <350 and >550 bp after merging were discarded. To identify chimeric sequences, a reference-based chimera detection step was conducted using the VSEARCH tool against the SILVA gold database^23,24^. Next, the sequence reads were clustered into operational taxonomic units (OTUs) using VSEARCH with a de novo clustering algorithm, with 97% sequence similarity as the threshold. The representative sequences of the OTUs were finally classified using the SILVA 128 database with UCLUST (parallel_assign_taxonomy_uclust.py script on QIIME version 1.9.1) and default parameters^25^.

### Tissue processing and flow cytometric analysis for ILC2s

Sinonasal tissues were minced using sterile scissors. The tissues were then strained through a 70-μm nylon mesh cell strainer into 50-ml polypropylene tubes containing PBS and 1% fetal bovine serum (FBS; MP Biomedicals, Aurora, OH, USA). Tissue pellets were obtained by centrifugation for 5 min at 600 × *g*.

For intracellular cytokine staining, a single cell suspension was stimulated with 25 ng/ml phorbol 12-myristate 13-acetate and 500 ng/ml ionomycin (Thermo Fisher Scientific) in the presence of Protein Transport Inhibitor Cocktail (Thermo Fisher Scientific) for 3 h at 37 °C. The Intracellular Fixation & Permeabilization Buffer Set (eBioscience, San Diego, CA, USA) was used for cell permeabilization, staining, and subsequent washing. The following antibodies were used at the concentrations recommended by the manufacturers: allophycocyanin (APC)-eFlour780-conjugated anti-CD45; fluorescein isothiocyanate-conjugated lineage markers (anti-CD1a, anti-CD11c, anti-CD14, anti-CD19, anti-CD34, anti-CD94, anti-CD123, anti-CD303 (201 A), anti-T-cell receptor (TCR) αβ, anti-TCRγδ, and anti-FcεRIa); eFluo450-conjugated anti-CD127; phycoerythrin (PE)-Cy7-conjugated anti-161; APC-conjugated anti-CRTH2; and PE-conjugated anti-IL-5 (Thermo Fisher Scientific). The fixable viability dye eFluor 506 (eBioscience) was added to exclude dead cells. Cells were fixed with 200 μl of 1% paraformaldehyde. Data were acquired using a FACSCanto II apparatus (BD Biosciences, Santa Clara, CA, USA) and were analyzed with FlowJo software (Tree Star, Ashland, OR, USA). IL-5-producing ILC2s were identified as lineage ^−^CD45^+^CD127^+^CD161^+^CRTH2^+^IL-5^+^ cells^26^. The prevalence of IL-5-producing ILC2s was calculated as the number of IL-5^+^ ILC2s divided by the total number of CD45^+^ cells.

### ELISA for cytokines and chemokines in human tissue homogenates

Fresh tissue was weighed and homogenized with 1 ml of a mixture containing Protease Inhibitor Cocktail (Roche, Basel, Switzerland), Phosphatase Inhibitor Cocktail (Roche), 150 mM sodium chloride, 1% Triton X-100, 0.5% sodium deoxycholate, 0.1% sodium dodecyl sulfate, 50 mM Tris-HCl, and 2 mM EDTA per 100 mg of tissue. The homogenates were centrifuged at 11,000 × *g* for 10 min at 4 °C. The supernatants were collected and stored at −80 °C until analysis. IL-5, IL-8, IL-13, interferon-gamma (IFN-γ), C–C motif chemokine (CCL)11, and CCL24 levels were measured using a DuoSet human ELISA kit (R&D Systems, Minneapolis, MN, USA) according to the manufacturer’s instructions.

### Statistical analyses

The demographic and clinical variables were compared using the Kruskal–Wallis test or Chi-square test. Standard ecological diversity indices (e.g., Chao1 and Shannon diversity) were assessed using the Kruskal–Wallis test. Beta diversity (distance between samples based on differences in OTUs present in each sample) was measured using principal coordinate analysis (PCoA) based on Bray–Curtis dissimilarity. A heat map was used to visualize ordination. Comparison of the relative abundance (RA) of OTUs between groups was performed using the Wilcoxon rank sum test.

Linear discriminant analysis (LDA) effect size (LEfSe) was used to determine genera that best characterized each group (control vs. eosinophilic CRSwNPs vs. noneosinophilic CRSwNPs). LEfSe uses LDA to estimate the effect size of each differentially rich feature^27^. An LDA score >3 and *P* < 0.05 were considered significant. Correlations were evaluated using Pearson’s correlation analysis. Statistical analyses were performed with the R software package. Statistical significance was evident as *P* < 0.05.

## Results

Eosinophilic CRSwNPs patients were older and had higher asthma incidence, polyp grade, Lund–Kennedy endoscopic score, and Lund–Mackay CT score than control and noneosinophilic CRSwNPs patients. Noneosinophilic CRSwNPs patients had a higher prevalence of purulent discharge and saline irrigation in the 6 months before surgery than eosinophilic CRSwNPs patients (Table 1).

**Table 1:** Preoperative demographic characteristics of subjects.

Characteristics	Control (*n* = 6)	Noneosinophilic CRSwNPs (*n* = 10)	Eosinophilic CRSwNPs (*n* = 21)	*P* value
Age (years)^a^	33.7 ± 11.4	37.3 ± 13.9	50.6 ± 13.5	0.011
Male (%)	66.7	70.0	66.7	0.982
Atopy (%)	0.0	30.0	47.6	0.091
Asthma (%)	0.0	0.0	38.1	0.020
Never smoker (%)	83.3	70.0	66.7	0.733
Purulent discharge (%)	0	80.0	66.7	0.004
Systemic antibiotics from 6 to 1 months before surgery (%)	0.0	38.1	40.0	0.178
Saline irrigation in 6 months before surgery (%)	0	50.0	4.8	0.003
Davos polyp classification^a^	0	3.5 ± 1.8	4.0 ± 1.6	<0.001
Lund–Kennedy endoscopic score^a^	0	9.3 ± 2.8	10.1 ± 2.4	<0.001
Lund–Mackay CT score^a^	0	15.6 ± 5.1	18.0 ± 3.9	<0.001
Preoperative SNOT-22 score^a^	40.2 ± 32.5	42.0 ± 22.7	42.3 ± 23.2	0.555
Blood eosinophils (/μl)^a^	240.9 ± 184.7	225.8 ± 230.1	386.6 ± 237.6	0.076
Serum total IgE (kU/l)^a^	35.4 ± 48.3	98.4 ± 65.3	226.6 ± 192.3	0.016

*SNOT* sinonasal outcome test.

^a^Mean ± standard deviation.

Sinus bacterial alpha diversity did not differ significantly among control, eosinophilic CRSwNPs, and noneosinophilic CRSwNPs subjects (Fig. E1a). At the phylum level, analysis of OTUs demonstrated a rich set of bacterial taxa in both control subjects and patients dominated by characteristic bacterial phyla that included *Firmicutes* (mainly *Staphylococci*), *Actinobacteria* (mainly *Corynebacterium*, *Bifidobacterium*, and *Propionibacterium* species), and *Proteobacteria* (mainly *Moraxella*, *Pseudomonas*, *Enterobacter*, and *Aggregatibacter*). Sinus bacteria did not differ at the phylum level between control subjects, eosinophilic CRSwNPs patients, and noneosinophilic CRSwNPs patients (Fig. E1b).

**Fig. 1:**
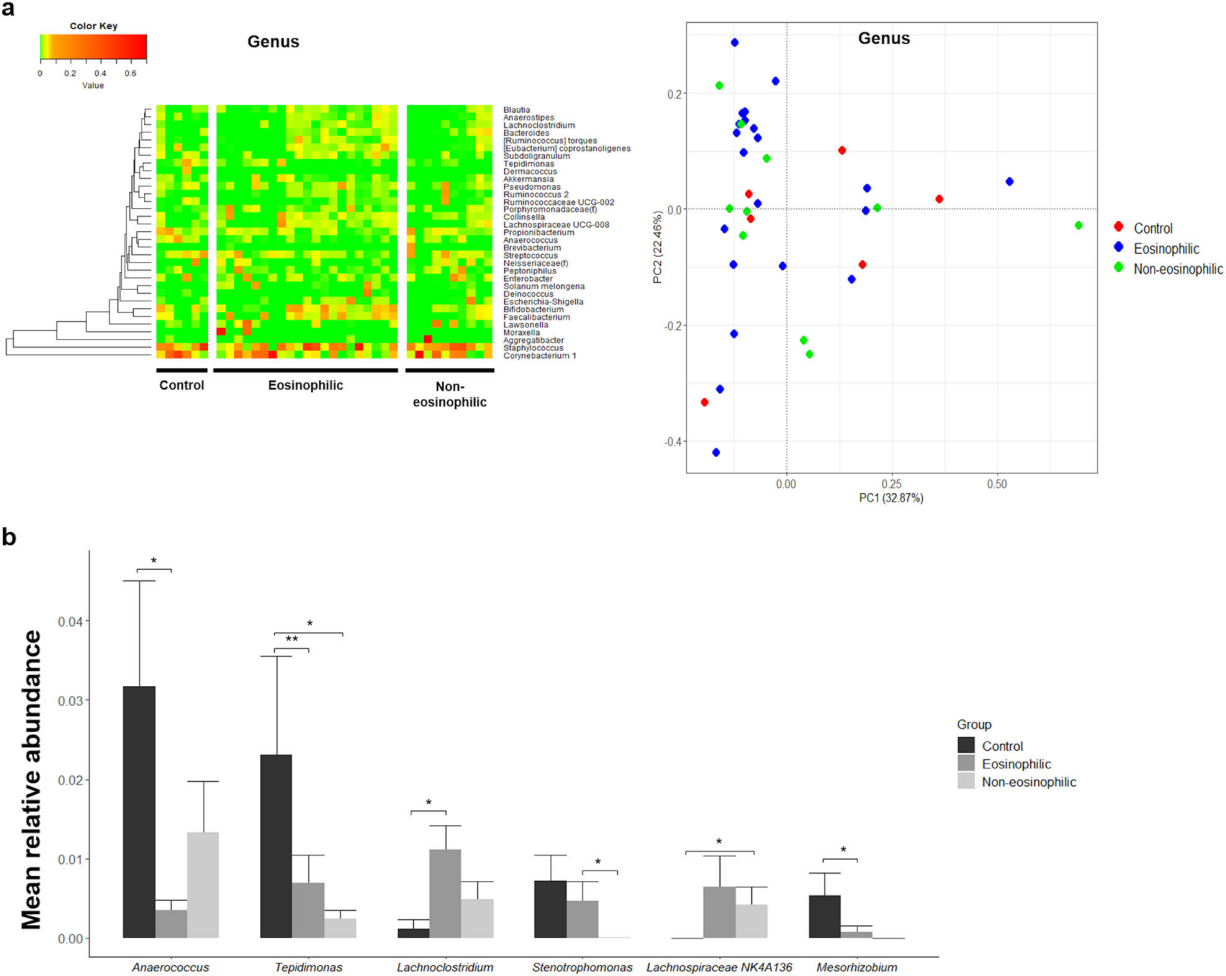
Significantly differentially abundant bacterial genera in the sinus among control subjects, patients with eosinophilic chronic rhinosinusitis with nasal polyps, and patients with noneosinophilic chronic rhinosinusitis with nasal polyps. **a** Differences in the composition of sinus bacteria between the three groups were identified by a heat map, although there was distinct clustering on the PCoA plot. **b** In patients with eosinophilic chronic rhinosinusitis with nasal polyps, the RAs of *Anaerococcus*, *Tepidimonas*, and *Mesorhizobium* were significantly decreased, whereas that of *Lachnoclostridium* was significantly increased compared with those in controls. *P < 0.05, **P < 0.01.

**Fig. 2:**
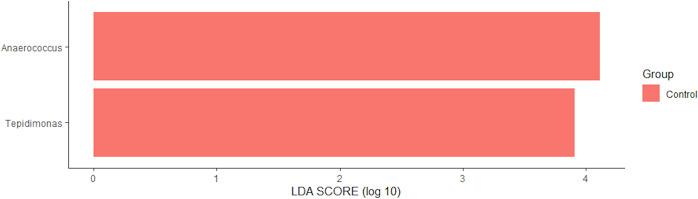
Linear discriminant analysis (LDA) showing distinct bacterial genera in the sinus. Significantly differentially abundant genera with *P* < 0.05 and LDA score >3 were observed.

However, sinus bacterial compositions were different at the genus level among control subjects, eosinophilic CRSwNPs patients, and noneosinophilic CRSwNPs patients (Fig. 1a). In eosinophilic CRSwNPs patients, the RAs of *Anaerococcus* and *Tepidimonas* were significantly decreased (*P* = 0.010 and *P* = 0.009, respectively), while that of *Lachnoclostridium* was significantly increased (*P* = 0.038) when compared with those in controls. The RA of *Stenotrophomonas* was significantly increased in eosinophilic CRSwNPs patients compared with that in noneosinophilic CRSwNPs patients (*P* = 0.038), the RA of *Lachnospiraceae NK4A136* was increased in noneosinophilic CRSwNPs patients compared with that in controls (*P* = 0.039), and the RA of *Mesorhizobium* was significantly decreased in eosinophilic CRSwNPs patients compared with that in controls (*P* = 0.012) (Fig. 1b). When LEfSe was used to compare bacterial abundance at the genus level, *Anaerococcus* and *Tepidimonas* were significantly enriched in control subjects (Fig. 2), similar to the results of the comparison between groups.

Correlations between immunological features, such as ILCs, cytokines, and chemokines, and distinct bacteria (*Anaerococcus*, *Tepidimonas*, *Lachnoclostridium*, *Stenotrophomonas*, and *Lachnospiraceae NK4A136*) were analyzed. The RA of *Lachnoclostridium* was significantly positively correlated with IL-5-producing ILC2 populations (*R* = 0.340, *P* = 0.049; Fig. E2) without a significant correlation with IL-5 levels (*R* = 0.188, *P* = 0.266). Furthermore, IL-5-producing ILC2 populations correlated with IL-5 (*R* = 0.351, *P* = 0.042) and CCL24 (*R* = 0.351, *P* = 0.042) levels in tissues. The RA of *Anaerococcus* showed a negative correlation with IL-5-producing ILC2 populations (*R* = −0.332, *P* = 0.055) and a significant negative correlation with IL-5 levels in tissues (*R* = −0.420, *P* = 0.010; Table 2). There were significant correlations between the levels of other cytokines and chemokines (IL-8, IL-13, IFN-γ, CCL11, and CCL24) and the RAs of *Anaerococcus* and *Lachnoclostridium*. The RAs of *Tepidimonas*, *Stenotrophomonas*, and *Lachnospiraceae NK4A136* were not correlated with ILCs, cytokines, and chemokines (all *P* > 0.05; Table E1).

**Table 2:** Correlation between sinus bacteria and IL5-producing group 2 innate lymphoid cells (ILC2s) and IL-5 levels in sinonasal tissue.

Genera	IL5-producing ILC2		IL-5	
	*R*	*P*	*R*	*P*
*Anaerococcus*	−0.332	0.055	−0.420	0.010
*Tepidimonas*	−0.119	0.502	−0.034	0.843
*Lachnoclostridium*	0.340	0.049	0.188	0.266
*Stenotrophomonas*	0.195	0.269	0.164	0.332
*Lachnospiraceae*	−0.045	0.801	0.054	0.751

**Fig. 3:**
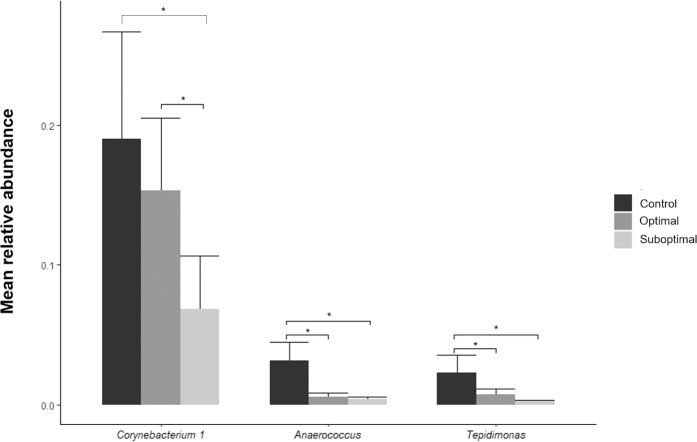
Significantly differentially abundant bacterial genera in the sinus according to the surgical outcome. *Corynebacterium*, *Anaerococcus*, and *Tepidimonas* were significantly less abundant in patients with suboptimal outcomes than in those with optimal outcomes and control subjects. *P < 0.05.

Patients with suboptimal outcomes exhibited significantly decreased abundances of *Corynebacterium* at the time of surgery compared with those of patients with optimal outcomes and controls (6.9% RA vs. 15.3% RA vs. 19.0% RA, *P* = 0.049). The RAs of *Anaerococcus* and *Tepidimonas* were also significantly decreased in patients with suboptimal outcomes compared with those in patients with optimal outcomes and control subjects (0.4% RA vs. 0.6% RA vs. 3.2% RA, *P* = 0.033 and 0.3% RA vs. 0.8% RA vs. 2.3% RA, *P* = 0.197, respectively; Fig. 3). Among patients with optimal outcomes, the RA of *Subdoligranulum* was increased in eosinophilic CRSwNPs patients compared with that in noneosinophilic CRSwNPs patients (0.9% RA vs. 0.0% RA, *P* = 0.026). Among patients with suboptimal outcomes, the RA of *Klebsiella* was increased in eosinophilic CRSwNPs patients compared with that in noneosinophilic CRSwNPs patients (0.3% RA vs. 0.0% RA, *P* = 0.045), while the RAs of *Gordonia* and *Ruminococcus gnavus* were decreased in eosinophilic CRSwNPs patients compared with those in noneosinophilic CRSwNPs patients (0.0% RA vs. 0.3% RA, *P* = 0.049 for each).

**Fig. 4:**
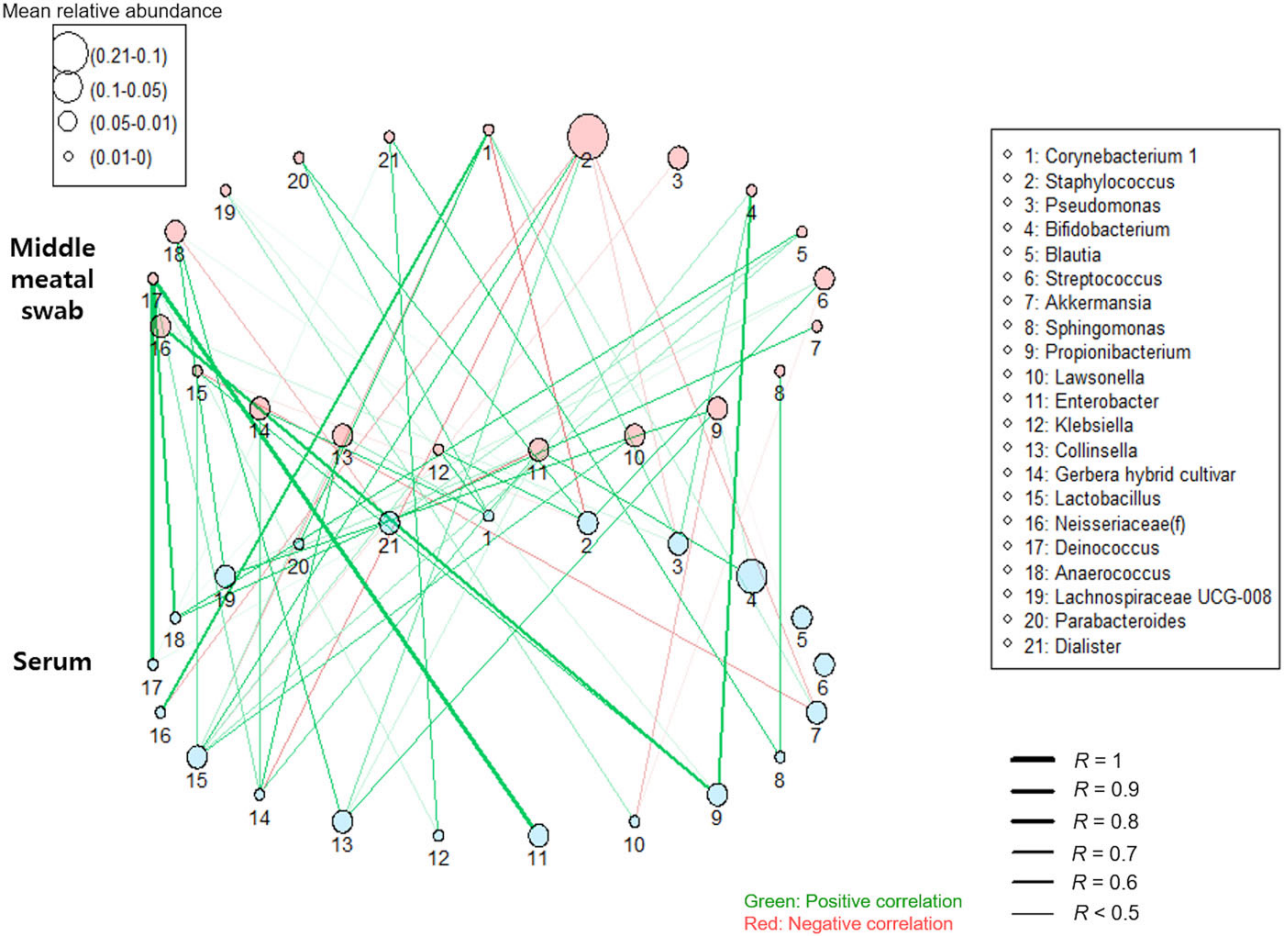
Correlation between sinus bacteria and serum extracellular vesicles. *Deinococcus*, *Sphingomonas*, and *Lactobacillus* among the sinus bacteria were positively correlated with serum extracellular vesicles.

A moderate-to-strong positive correlation between sinus bacteria and serum EVs was evident for *Deinococcus* (*R* = 0.921, *P* < 0.001), *Sphingomonas* (*R* = 0.599, *P* = 0.009), and *Lactobacillus* (*R* = 0.423, *P* = 0.009). Moreover, there was a strong positive correlation between *Deinococcus* in sinus bacteria and *Enterobacter* in serum EVs (*R* = 0.929, *P* < 0.001), between *Corynebacterium* in sinus bacteria and *Neisseriaceae* in serum EVs (*R* = 0.826, *P* < 0.001), between *Bifidobacterium* in sinus bacteria and *Propionibacterium* in serum EVs (*R* = 0.683, *P* = 0.002), between *Staphylococcus* in sinus bacteria and *Lactobacillus* in serum EVs (*R* = 0.599, *P* = 0.009), and between *Anaerococcus* in sinus bacteria and *Lachnospiraceae.UCG.008* in serum EVs (*R* = 0.547, *P* = 0.019; Fig. 4).

## Discussion

Various microbes, including bacteria, viruses, and fungi, thrive in the human body in a symbiotic relationship^28^. Many microbiota-related studies have been performed with the knowledge that the microbiota strongly influences human health and diseases^29^. These studies have been performed not only for the gut but also for the respiratory tract, which is one of the entry routes of bacteria into the body. Bacteria have been implicated in the pathogenesis of CRS, and staphylococcal superantigen, immune barrier, and biofilm hypotheses have been proposed^30–32^. However, the exact roles of bacteria in CRS remain controversial.

In the present study, eosinophilic CRSwNPs patients showed significantly lower RAs of *Anaerococcus* and *Tepidimonas* and a higher RA of *Lachnoclostridium* at the genus level than controls and noneosinophilic CRSwNPs patients. LEfSe also showed *Anaerococcus* and *Tepidimonas* enrichment in controls. The clinical and demographic variables were different among controls, eosinophilic CRSwNPs patients, and noneosinophilic CRSwNPs patients. These variables might affect bacterial composition. Eosinophilic CRSwNPs patients had higher asthma incidence and clinical severity scores but lower purulence incidence and saline irrigation than noneosinophilic CRSwNPs patients. These findings indicate that asthma, clinical severity, and purulence may be associated with significant alterations in bacterial composition, similar to the findings of a previous study^17^, although a direct relationship was not analyzed. The three groups showed similar rates of smoking, systemic antibiotic administration before surgery, and saline irrigation. Given that these factors were not associated with distinct alterations in sinonasal bacteria^33–35^, their effects on the microbiome might not be significant.

In one study, when a CRS patient cluster was divided into distinct subgroups, each subgroup was associated with a distinct metabolism and host immune response^7^. The immunologic features associated with the nature of the sinus microbiota can partially explain CRS heterogeneity and can be used as a basis for establishing a customized treatment strategy. In our study, the IL-5-producing ILC2 population showed a significant positive correlation with the RA of *Lachnoclostridium* but a negative correlation with the RA of *Anaerococcus*. The observation that IL-5-producing ILC2 populations correlated with the mediators of eosinophilic inflammation, such as IL-5 and CCL24 levels in tissues, might explain the RA of *Lachnoclostridium* and the reduction in *Anaerococcus* abundance in eosinophilic CRSwNPs patients. Increased *Lachnoclostridium* abundance with type 2 responses and gut dysbiosis was suggested as a mechanism of food allergy induction during consumption of a high-fat diet^36^. Knowledge about the association between *Anaerococcus* and the immune response is limited. Given that *A. prevotii* is frequently identified from vaginal discharges and ovarian, peritoneal, sacral, or lung abscesses^37^, *Anaerococcus* may be related to the type 1 response.

CD4^+^ T helper (Th) cells play an important role in coordinating adaptive immune responses. Endotypes of CRS are differentiated mainly on the basis of Th cells other than ILCs, which produce cytokines and chemokines, leading to the accumulation of eosinophils and neutrophils^38^. The bacteria are associated with the endotypes of CRS in relation to cytokines produced by Th cells. Gram-negative bacteria were isolated more frequently in IL-5/IL-17/IFN-γ-negative NPs patients, whereas gram-positive bacteria were more associated with IL-5-positive NPs patients^39^. Moreover, depletion of commensal bacteria using oral broad-spectrum antibiotics increases serum IgE levels and exacerbates basophil-mediated Th2 cell responses and allergic inflammation in the airway^40^.

Bacterial diversity and composition were previously suggested as predictors of surgical outcome. In 27 patients with CRS, more diverse bacterial communities and enriched *Actinobacteria* at the phylum level and *Corynebacterium* at the genus level showed optimal outcomes than suboptimal outcomes^17^. Similarly, in our study, patients with optimal outcomes were more enriched for the genera *Corynebacterium*, *Anaerococcus*, and *Tepidimonas* at the time of surgery than those with suboptimal outcomes and less enriched than controls.

EVs circulate through the blood and directly and/or indirectly communicate with tissues and organs^10^. Blood bacterial EVs were suggested to be useful for metagenome analysis of the bodily microbiota in preterm birth and a mouse model of Alzheimer’s disease^41,42^. In this study, *Deinococcus*, *Sphingomonas*, and *Lactobacillus* among the sinus bacteria were positively correlated with serum EVs. Individual bacteria in the middle meatus and serum were also correlated. These correlations suggested that sinus bacteria might communicate with blood bacterial EVs. Although direct causality between sinus bacteria and blood bacterial EVs and the mechanism of the latter’s formation have not been proven, *Sphingomonas* may be potentially meaningful in terms of sinus bacteria and blood bacteria communication. The *Sphingomonadaceae* family was found to be abundant in the urine-derived EVs of children with asthma and chronic rhinitis compared with that in EVs of controls^43^. *Sphingomonas* species in the airway were associated with increased airway hyperresponsiveness to methacholine, positively correlated with eosinophil cationic protein levels, and strongly and positively correlated with serum IgE levels and blood and sputum eosinophil counts in patients with asthma^44–46^. This association may be explained by glycosphingolipid, a cell membrane component of *Sphingomonas* that activates natural killer T cells producing IL-4 and IL-13^47–49^. Further research is needed on the association of *Sphingomonas* between the sinus and serum in relation to type 2 immune responses and eosinophilic CRSwNPs. Another potentially interesting bacterium is *Lactobacillus*; its RA in the sinus is less than that of other bacteria, but its sinus RA is positively correlated with its RA in serum EVs. Considering that *Lactobacillus* has been suggested as a potential protective species against allergens and virus infection^50,51^, deficiency in *Lactobacillus* in the sinus and blood may be associated with the pathophysiology of CRS.

The current study included a small number of subjects. However, the observed relationship between immunological characteristics in terms of ILCs and bacteria in the sinonasal cavity in CRSwNPs patients is a novel finding. Because there was large variation in the bacterial composition of the sinonasal cavity between individuals, similar to that in a previous study^7^, clustering based on the clinical characteristics was not distinct. Bacterial metabolites were not investigated in this study. A previous study demonstrated that the *Streptococcaceae* dominant group, *Pseudomonadaceae* dominant group, *Corynebacteriaceae* dominant group, and *Staphylococcaceae* dominant group encoded enriched gene pathways, including ansamycin biosynthesis, tryptophan metabolism, the two-component response system virulence pathway, and the peroxisome proliferator-activated receptor gamma signaling pathway, respectively. These metabolites may be involved in the immune responses of nasal polyp tissues^7^. Therefore, the effect of the metabolites of *Lachnoclostridium* and *Anaerococcus* on ILC2s in NP tissues should be further investigated.

Finally, this study showed that the sinus microbiota, particularly *Anaerococcus* and *Lachnoclostridium* in CRSwNPs patients, was associated with eosinophilic inflammation and IL-5-producing ILC2s. The abundances of the genera *Corynebacterium*, *Anaerococcus*, and *Tepidimonas* were related to surgical outcome. Furthermore, some sinus bacteria were correlated with blood bacterial EVs. Therefore, understanding the sinus microbiota of individuals could be the basis for effective treatment of CRS and for predicting prognosis.

## Supplementary information

Online Supplementary Information
