# Supplementary material for: Association between the sinus microbiota with eosinophilic inflammation and prognosis in chronic rhinosinusitis with nasal polyps
Source: Exp Mol Med. 2020 Jun 29;52(6):978–87. doi: 10.1038/s12276-020-0458-1 (PMC7338545; doi:10.1038/s12276-020-0458-1)
Supplement: Supplementary file 1 — Online Supplementary Information [file 12276_2020_458_MOESM1_ESM.docx]

**Online Supplementary Information**

| Genera | IL-8 | | IL-13 | | IFN- γ | | CCL11 | | CCL24 | |
| --- | --- | --- | --- | --- | --- | --- | --- | --- | --- | --- |
|  | *R* | *P* | *R* | *P* | *R* | *P* | *R* | *P* | *R* | *P* |
| *Anaerococcus* | -0.131 | 0.441 | -0.067 | 0.695 | 0.108 | 0.538 | 0.114 | 0.500 | -0.254 | 0.129 |
| *Tepidimonas* | -0.211 | 0.211 | -0.054 | 0.752 | 0.175 | 0.313 | 0.182 | 0.280 | -0.084 | 0.620 |
| *Lachnoclostridium* | -0.128 | 0.452 | 0.086 | 0.614 | 0.106 | 0.544 | 0.080 | 0.639 | 0.300 | 0.072 |
| *Stenotrophomonas* | -0.183 | 0.278 | 0.036 | 0.831 | -0.036 | 0.831 | -0.104 | 0.542 | -0.087 | 0.609 |
| *Lachnospiraceae* | -0.008 | 0.960 | -0.047 | 0.782 | 0.008 | 0.962 | 0.174 | 0.302 | 0.197 | 0.243 |

**Table E1.** Correlation between sinus bacteria and cytokines and chemokines levels in sinonasal tissue


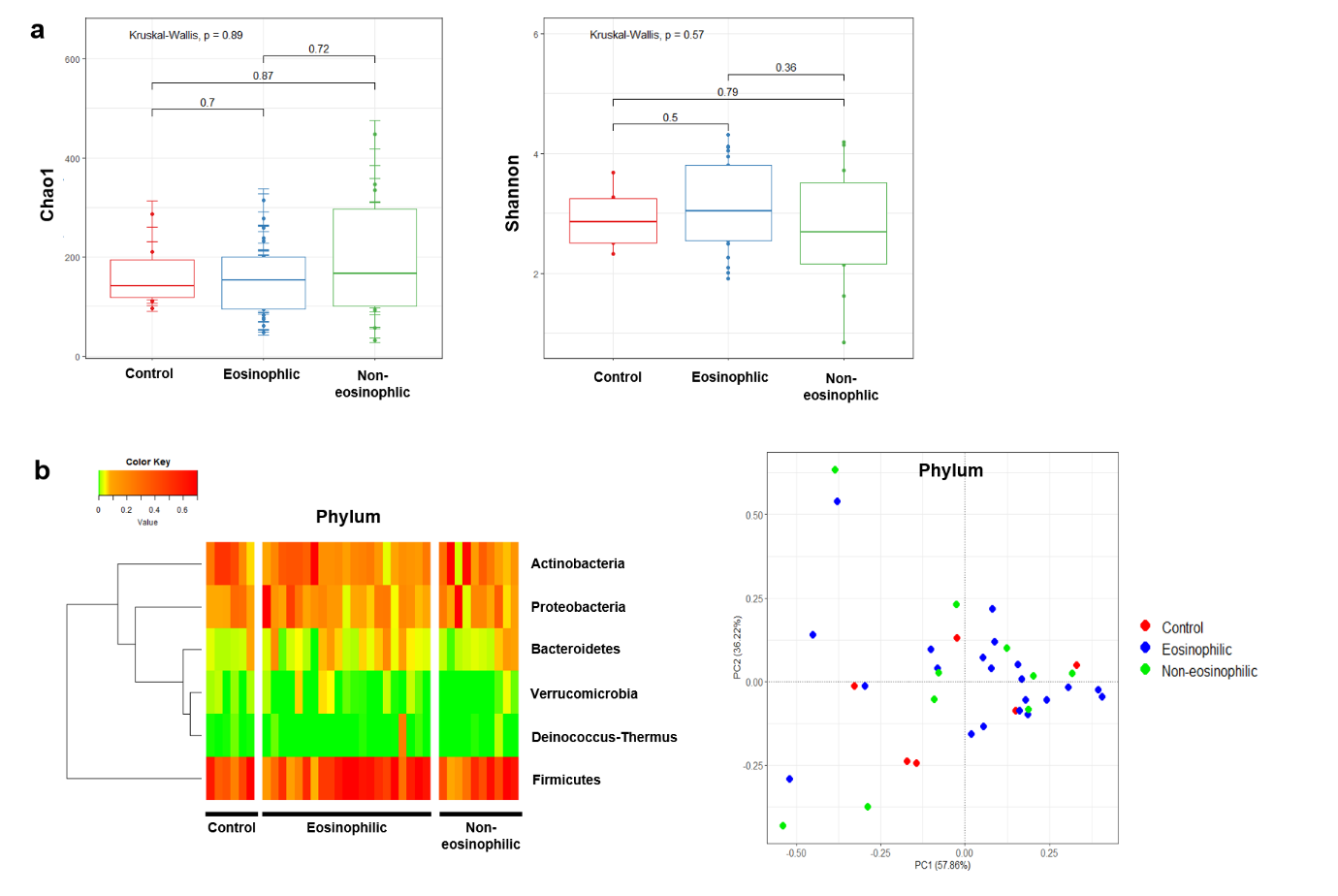


**Figure E1**. Sinus bacterial biodiversity at the phylum level. (a) There were no significant differences in Chao1 and Shannon indexes of sinus bacteria among control subjects, patients with eosinophilic chronic rhinosinusitis with nasal polyps, and patients with non-eosinophilic chronic rhinosinusitis with nasal polyps. (b) At the phylum level, there were no significant differences in RA of sinus bacteria among control subjects, eosinophilic chronic rhinosinusitis with nasal polyps patients, and non-eosinophilic chronic rhinosinusitis with nasal polyps patients.

**
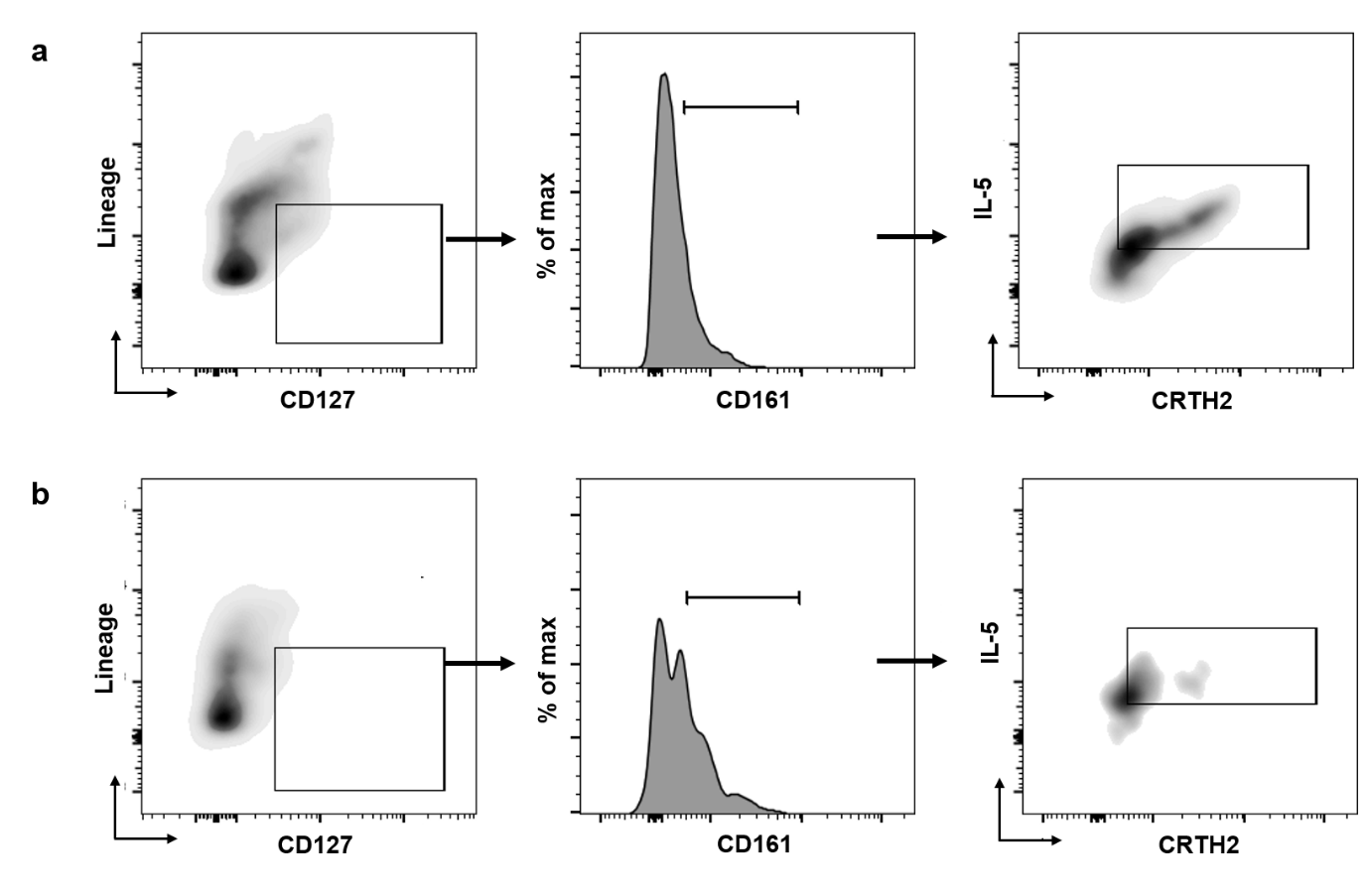
**

**Figure E2.** Differences in IL-5-producing group 2 innate lymphoid cells (ILC2) between *Lachnoclostridium-*enriched patients with eosinophilic chronic rhinosinusitis with nasal polyps (a) and *Lachnoclostridium-*deficient controls (b). Cells were first gated for live (Fixable Viability Dye eFluor 506-negative) and small/non-granular (FSC^low^SSC^low^) leukocytes (CD45^+^), and IL-5-producing ILC2 were gated for lineage^-^CD127^+^CD161^+^CRTH2^+^IL-5^+^.
